# Protein structure determination by electron diffraction using a single three-dimensional nanocrystal

**DOI:** 10.1107/S2059798317010348

**Published:** 2017-08-15

**Authors:** M. T. B. Clabbers, E. van Genderen, W. Wan, E. L. Wiegers, T. Gruene, J. P. Abrahams

**Affiliations:** a Center for Cellular Imaging and NanoAnalytics (C-CINA), Biozentrum, Basel University, Mattenstrasse 26, CH-4058 Basel, Switzerland; b Department of Biology and Chemistry, Paul Scherrer Institut (PSI), CH-5232 Villigen PSI, Switzerland; c Department of Materials and Environmental Chemistry, Stockholm University, SE-106 91 Stockholm, Sweden; d Leiden Institute of Physics, Leiden University, Niels Bohrweg 2, 2333 CA Leiden, The Netherlands; e Leiden Institute of Biology, Leiden University, Sylviusweg 72, 2333 BE Leiden, The Netherlands

**Keywords:** electron crystallography, protein nanocrystals, hybrid pixel detector

## Abstract

Three-dimensional nanometre-sized crystals of macromolecules currently resist structure elucidation by single-crystal X-ray crystallography. Here, a single nanocrystal with a diffracting volume of only 0.14 µm^3^, *i.e.* no more than 6 × 10^5^ unit cells, provided sufficient information to determine the structure of a rare dimeric polymorph of hen egg-white lysozyme by electron crystallography. This is at least an order of magnitude smaller than was previously possible. The molecular-replacement solution, based on a monomeric polyalanine model, provided sufficient phasing power to show side-chain density, and automated model building was used to reconstruct the side chains. Diffraction data were acquired using the rotation method with parallel beam diffraction on a Titan Krios transmission electron microscope equipped with a novel in-house-designed 1024 × 1024 pixel Timepix hybrid pixel detector for low-dose diffraction data collection. Favourable detector characteristics include the ability to accurately discriminate single high-energy electrons from X-rays and count them, fast readout to finely sample reciprocal space and a high dynamic range. This work, together with other recent milestones, suggests that electron crystallography can provide an attractive alternative in determining biological structures.

## Abstract 2

A single three-dimensional protein nanocrystal was used for structure determination by electron diffraction. Data were acquired using the rotation method with a Timepix hybrid pixel detector for low-dose data acquisition.

## 1. Introduction   

Electron crystallography can be used for structure determination of macromolecules from crystalline samples. Originally, the method concentrated on diffracting and imaging two-dimensional crystals (Raunser & Walz, 2009 ▸; Stahlberg *et al.*, 2015 ▸), and resulted in important structures of membrane proteins (Unwin & Henderson, 1975 ▸; Gonen *et al.*, 2005 ▸). Electron diffraction of three-dimensional crystals allowed the structure solution of organic and inorganic samples (Vain­shtein, 1964 ▸; Dorset, 1995 ▸; Weirich *et al.*, 1996 ▸; Mugnaioli *et al.*, 2009 ▸; Kolb *et al.*, 2010 ▸; Gorelik *et al.*, 2012 ▸; Zou *et al.*, 2011 ▸; Guo *et al.*, 2015 ▸). Crystallographic data are most efficiently collected by continuously rotating the crystal (Dauter, 1999 ▸). The rotation method has been the standard approach for data collection in protein crystallography for the last four decades (Arndt & Wonacott, 1977 ▸). In electron crystallography, alignment of the crystal with the rotation axis is not always straightforward and the rotation stages are not always as accurate as desired, which prompted the enhancement of the method using either conical beam precession (Vincent & Midgley, 1994 ▸; Kolb *et al.*, 2007 ▸, 2008 ▸; Gemmi *et al.*, 2013 ▸) or beam tilt (Zhang *et al.*, 2010 ▸; Wan *et al.*, 2013 ▸; Yun *et al.*, 2015 ▸). Recently, continuous three-dimensional data collection from protein nanocrystals was accomplished (Nederlof *et al.*, 2013 ▸). The first protein structure of a micrometre-sized crystal was determined soon after, using discrete rotation steps (Shi *et al.*, 2013 ▸). More recently, continuous rotation became the preferred method in protein electron crystallography (Nannenga, Shi, Leslie *et al.*, 2014 ▸; Nannenga, Shi, Hattne *et al.*, 2014 ▸; Yonekura *et al.*, 2015 ▸). The attractiveness of electron crystallography for macromolecular samples is further encouraged by the observation that a large fraction of seemingly failed crystallization attempts contain nanocrystals (Stevenson *et al.*, 2014 ▸, 2016 ▸). Nanocrystals may also contain fewer defects than micrometre-sized crystals and lead to better data quality (Cusack *et al.*, 1998 ▸; de la Cruz *et al.*, 2017 ▸).

The electrostatic scattering potential map, which is the basis for model building, is calculated by a Fourier transform of the phased structure-factor amplitudes and assumes kinematic scattering. Dynamic scattering affects (Cowley & Moodie, 1957 ▸; Dorset *et al.*, 1992 ▸; Glaeser & Downing, 1993 ▸), but does not prevent, structure solution using electron diffraction data (Dorset, 1995 ▸; Glaeser & Downing, 1993 ▸; Palatinus *et al.*, 2017 ▸). In the presence of multiple scattering, the diffraction data can no longer be interpreted using a purely kinematic approximation where *I*(*hkl*) ∝ |*F*(*hkl*)|^2^. Structure refinement against electron diffraction data using dynamical scattering theory (Jansen *et al.*, 1998 ▸; Palatinus, Petříček *et al.*, 2015 ▸; Palatinus, Corrêa *et al.*, 2015 ▸; Palatinus *et al.*, 2017 ▸) is not yet available for protein crystals. However, if the crystalline sample is sufficiently thin then this ensures that the measured data are predominantly kinematic and should not hamper structure solution too severely (Cowley & Moodie, 1957 ▸). The small crystal volume directly affects data acquisition; smaller crystals require longer exposure to obtain the same signal-to-noise ratio (SNR) as larger crystals, which results in more radiation damage. Radiation damage is a major limiting factor in the study of macromolecules (Henderson, 1995 ▸; Owen *et al.*, 2006 ▸); thus, diffraction data need to be collected under low-dose cryoconditions and sensitive, low-noise electron detection is imperative.

Previously, we used a single quad Medipix detector (Georgieva *et al.*, 2011 ▸; Nederlof *et al.*, 2013 ▸) and a Timepix detector (van Genderen *et al.*, 2016 ▸) of 512 × 512 pixels (55 × 55 µm pixel size). For very well ordered crystals this detector size is sufficient for resolving up to 50 orders of diffraction. However, for protein crystals with larger unit cells, preventing overlap between adjacent Bragg spots may impose a (virtual) detector distance that limits the resolution of the diffraction patterns^1^. Tiling of multiple Timepix quad detectors to give larger arrays can overcome these difficulties. Therefore, we developed a novel in-house-designed 1024 × 1024 pixel Timepix hybrid pixel detector (55 × 55 µm pixel size).

Detector features that are of particular interest for electron diffraction are the absence of readout noise, a high dynamic range and the ability to distinguish between the signal from diffracted electrons and that from the high X-ray background that is inherently present in any TEM (Georgieva *et al.*, 2011 ▸; Nederlof *et al.*, 2013 ▸; van Genderen *et al.*, 2016 ▸). These features require a counting detector, a concept that has recently also been introduced in monolithic and CMOS detectors. Hybrid pixel detectors (such as the one employed here) only count high-energy electron hits in counting mode if the energy deposited in the silicon sensor layer for a single pixel is higher than a user-defined threshold during a clock cycle (Llopart *et al.*, 2002 ▸, 2007 ▸). This allows a linear detection range of more than 10^6^ electrons per pixel per second in counting mode. Monolithic and CMOS detectors count after the frame has been read out. So, for these detectors, the dynamic range per pixel in counting mode cannot exceed about one tenth of the number of frames that can be read out per second. This dynamic range is many orders of magnitude smaller than the dynamic range of hybrid pixel detectors.

Monolithic detectors are also more radiation-sensitive than hybrid pixel detectors because the electrons directly hit the integrating readout electronics of the detector. Since electron diffraction data can have spikes of high intensity at low resolution and in Bragg peaks, monolithic detectors are currently not used for measuring electron diffraction data. However, in hybrid pixel detectors the high-energy electrons are stopped by the silicon sensor layer that is bump-bonded to the counting and integration electronics (McMullan *et al.*, 2007 ▸, 2009 ▸; Faruqi & McMullan, 2011 ▸). The integrating electronics of CMOS detectors can be shielded by a phosphor, at the expense of an increased point spread. Thus, hybrid pixel detectors sacrifice pixel size to achieve radiation hardness, a high dynamic range and megahertz counting modes. Pixel size is less important in diffraction data acquisition than in imaging, since the resolution of the data is not determined by the level of detail on the detector but by the number of diffraction orders that can be resolved (Nederlof *et al.*, 2013 ▸).

Here, we present structure determination from a very thin single protein nanocrystal with a diffracting volume of only 0.14 µm^3^. Diffraction data were acquired using the rotation method on a novel Timepix hybrid pixel detector electron diffraction camera designed for electron crystallography. Standard data-processing procedures and software as commonly used in macromolecular X-ray crystallography were adopted for electron diffraction data with minor adaptations. We discuss instrumentation and data acquisition throughout structure solution, model building and refinement.

## 2. Methods   

### 2.1. Data acquisition   

**Figure 1:**
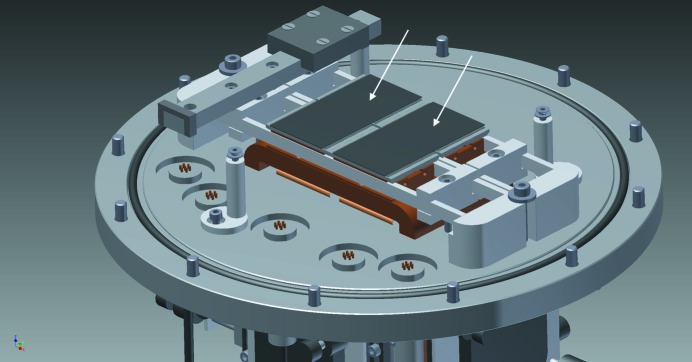
The flange design of the camera housing, including the Timepix hybrid pixel detector in the centre (Supplementary Fig. S1). The tiled detector assembly holds four Timepix quads (512 × 512 pixels each). The dark grey top layers pointed out by the arrows represent the sensitive silicon layers of a pair of Timepix quads and the light grey slabs below represent the chip board. The gaps between the chips are necessary to accommodate the wire bonds to the readout boards.

Electron diffraction data were acquired on an FEI Talos Arctica TEM (Center for Cellular Imaging and NanoAnalytics, Basel, Switzerland) and an FEI Titan Krios TEM (NeCEN, Leiden, The Netherlands). Both microscopes were equipped with a Timepix hybrid pixel detector (1024 × 1024 pixels, 55 × 55 µm pixel size). We developed a prototype of such a tiled detector camera of 2 × 2 Timepix quad detectors (Supplementary Fig. S1), which gave an effective array of 1024 × 1204 pixels (Fig. 1 ▸). The Timepix quad cannot be abutted without gaps of ∼35 pixels (horizontal) and ∼175 pixels (vertical). The former gap is imposed by the sensitive silicon layer being slightly larger than the pixel array, and the latter is imposed by the presence of the readout wire bonds on opposing sides of the detector chip.

Because high electron fluxes may be focused in Bragg spots, the energy of the incident electron should be completely deposited in the sensor layer to prevent any damage to the readout ASIC that is underneath. For 200 and 300 keV electrons the potential scattering distances are approximately 225 and 450 µm, respectively (McMullan *et al.*, 2007 ▸, 2009 ▸; Faruqi & McMullan, 2011 ▸). For the prototype, we used a 300 µm sensitive silicon layer. A thicker sensitive layer was considered which would allow the use of 300 keV electrons. However, because of the perpendicular impact of a 300 keV incident electron on the detector, on average the first pixel and the last pixel of its track receive the highest deposited dose. This means that at the energy threshold used for each pixel (∼60 keV), the electron is counted once (70%) or twice (30%) (McMullan *et al.*, 2007 ▸, 2009 ▸; Faruqi & McMullan, 2011 ▸). This means that the Bragg spot is spread out over a larger area. To reduce this effect, we opted for 200 keV electrons.

Hen egg-white lysozyme nanocrystals were prepared as described previously (Nederlof *et al.*, 2013 ▸). The microscope was operated at 200 kV and aligned for diffraction with a parallel beam that had a diameter of 2.0 and 1.7 µm in microprobe mode for the Talos Arctica and Titan Krios TEMs, respectively. EM grids were scanned for nanocrystals in imaging mode at 4k–10k magnification. Once a suitable crystal had been found, the crystal was centred on the rotation axis and the beam was centred on the crystal. Diffraction data were collected using the rotation method (Arndt & Wonacott, 1977 ▸), with continuous crystal rotation and shutterless data acquisition (Hasegawa *et al.*, 2009 ▸). A constant rotation of the goniometer was set using the *TADui* (FEI) and *TEMspy* (FEI) interfaces of the Talos Arctica and the Titan Krios, respectively. Independently, a fixed frame-exposure time was set with the *SoPhy* software (Amsterdam Scientific Instruments) for controlling the detector readout. Hence, each frame received the same electron dose and captured a constant rotation increment, as in the rotation method for X-ray crystallography. Data sets were collected with different fixed frame-exposure times (Supplementary Table S1). The dead time of the detector during readout amounted to 4–10% of the exposure time. During data acquisition, the dose rate on the Talos Arctica was ∼0.017 e^−^ Å^−2^ s^−1^. The electron flux on the Titan Krios was approximately 20 million electrons per second, amounting to a dose rate of ∼0.08 e^−^ Å^−2^ s^−1^ on the crystal (Supplementary Table S1).

### 2.2. Data processing   

**Figure 2:**
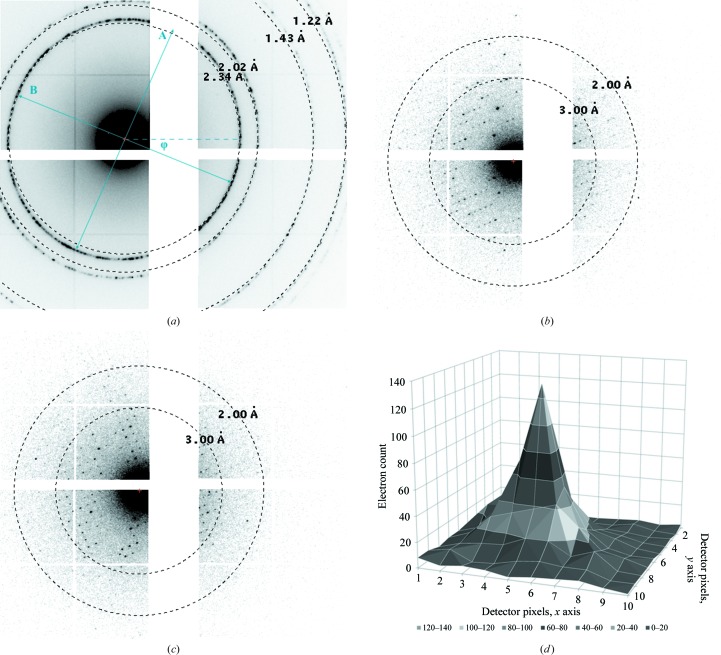
Electron diffraction data acquisition. (*a*) Measured powder pattern of an aluminium diffraction standard after correcting for the tiling offsets of the Timepix quad ASICs. An elliptical distortion can be observed with a deviation of 1.043 (= *A*/*B*) at an angle of φ = 21.3°. Diffraction from the single lysozyme crystal summed over 1.0° of rotation (*b*) from −17.0° to −16.0° and (*c*) from −6.0 to −5.0°. Crosses on individual quads are owing to corrections for larger border pixels as described by Nederlof *et al.* (2013 ▸) and van Genderen *et al.* (2016 ▸); these pixels were not taken into account for processing of the protein diffraction data. Note that owing to the radiation hardness of the detector, no backstop was required. Resolution rings were plotted with *ADXV* (http://www.scripps.edu/tainer/arvai/adxv.html). (*d*) A typical spot profile of a high-intensity peak at 16.33 Å resolution recorded on a single frame with an angular increment of 0.076° per frame at a dose rate of ∼0.01 e^−^ Å^−2^ per frame, shown in a 10 × 10 pixel array with 0.055 × 0.055 mm pixel size.

Output frames from the tiled detector were interpolated on an orthogonal grid and converted to PCK format (Abrahams, 1993 ▸) based on the positioning and orientation of the four individual Timepix quads (Figs. 1 ▸ and 2 ▸). We observed a small but significant elliptical distortion from powder diffraction patterns of an aluminium diffraction standard both before and after acquiring data. The distortion could not be modelled by a detector tilt. We determined the magnitude and orientation of the distortion (Fig. 2 ▸ *a*). Correction tables for *XDS* were generated by first creating a fake brass-plate pattern based on the distortion parameters using the program *geocorr.f*90 kindly provided by Dr Wolfgang Kabsch. The calculated geometric correction tables were used with the PILATUS template from *XDS*, with keywords X-GEO_CORR and Y-GEO_CORR (Kabsch, 2010 ▸).

The effective detector distance was calibrated using aluminium powder diffraction patterns after correcting for the elliptical distortion (Fig. 2 ▸ *a*). The orientation of the rotation axis was initially estimated by identifying reflections close to the rotation axis, which have a wider rocking curve. The angular frame width was assumed to be constant and was determined by dividing the total rotation range by the number of frames. Data were processed with *XDS* (Kabsch, 2010 ▸). Since the unit-cell parameters are unusual for lysozyme, and the quality indicators of electron diffraction data are very different to those for X-ray diffraction data, we confirmed the experimental parameters with *RED* (Wan *et al.*, 2013 ▸), which enables the quick, routine inspection of electron diffraction patterns in three-dimensional reciprocal space. After applying corrections for the elliptical distortion, *XDS* found the unit-cell dimensions with sufficient accuracy for data processing. Without applying these corrections, the elliptical distortion was too large for *XDS* to home in on the correct unit cell. The rotation-axis parameters were refined during data integration. The angular frame width was refined by minimizing the deviation of the unit-cell angles from an orthorhombic cell (Supplementary Table S1). With this, *XDS* suggested Laue group *mmm*, consistent with space group *P*2_1_2_1_2 (Supplementary Tables S2 and S3).

### 2.3. Structure solution   

Data sets were scaled with *XSCALE* (Kabsch, 2010 ▸), converted to MTZ format with *POINTLESS* (Evans, 2006 ▸) and merged with *AIMLESS* (Evans & Murshudov, 2013 ▸). Structure-factor amplitudes were obtained with *TRUNCATE* (Winn *et al.*, 2011 ▸). A polyalanine model of tetragonal lysozyme (PDB entry 2ybl; De la Mora *et al.*, 2011 ▸) was created using *CHAINSAW* (Winn *et al.*, 2011 ▸). The polyalanine monomer was used in a search in all orthorhombic primitive Sohncke groups in molecular replacement (MR) with *Phaser* (McCoy *et al.*, 2007 ▸). The Matthews coefficient suggested that the crystal contained two monomers per asymmetric unit, and *Phaser* unequivocally identified the rotation and translation parameters of both monomers and confirmed the space group as *P*2_1_2_1_2. Side chains were placed by automated model building with *Buccaneer*/*REFMAC*5 (Cowtan, 2006 ▸; Murshudov *et al.*, 2011 ▸). For the merged data three side chains were missing after autobuilding, although in all three instances clear difference potential was observed in the map (Supplementary Fig. S3). Thus, after inspecting the model and map these three missing residues were fitted using *Coot* (Emsley *et al.*, 2010 ▸). We did not further enhance the models by manual rebuilding in order to evaluate the extent to which refinement was able to correct errors in the model.

### 2.4. Refinement   

The model was optimized using *PDB_REDO* (Joosten *et al.*, 2014 ▸), in which electron scattering factors were set by placing ‘EXPDTA ELECTRON CRYSTALLOGRAPHY’ into the PDB header. The model was then refined with *REFMAC*5 (Murshudov *et al.*, 2011 ▸) using NCS restraints. To ensure convergence, the input model was refined for 1000 cycles using *REFMAC*5 (Supplementary Fig. S4). Electron scattering factors were set in *REFMAC*5 with the keyword ‘SOURCE ELECTRON MB’. To calculate the map coefficients, *REFMAC*5 was set to not restore unobserved reflections with the keyword ‘MAPC FREE EXCLUDE’.

We validated refinement in *REFMAC*5 with *R*_complete_ instead of *R*_free_. When considering data sets with less than about 10 000 unique reflections, as is the case for our data, calculating *R*_complete_ is preferred (Brünger, 1997 ▸). The *R*_complete_ validation method allows all reflections to be used in refinement, and thus our *R*_work_ is equivalent to *R*1. *R*1 defines how well the model explains all observed reflections. Like *R*_work_, it is likely to be affected by model bias. *R*_complete_ was calculated afterwards according to standard procedures with a 0.2% test-set size (Luebben & Gruene, 2015 ▸). Briefly, all nonmeasured observations were first removed from the reflection file with *SFTOOLS* (Winn *et al.*, 2011 ▸). 500 separate, non-overlapping and unique test sets were then randomly created with *FREERFLAG* (Winn *et al.*, 2011 ▸), each containing 0.2% of the observed structure-factor amplitudes. Thus, when combined, these test sets represent all data. 500 independent refinements were then performed until convergence, each time omitting a different test set. Each refinement started with the same (final) model from which *R*1 had been calculated. After each of the 500 validation refinement cycles had converged, the values of *F*_c_ were calculated from the resulting model. Only *F*_c_(*h*) values corresponding to reflections that had been omitted from that particular cycle (and thus were not biased by that cycle) were extracted. All these extracted reflections from each of the 500 independent refinement cycles were then combined into a single reflection file representing the unbiased *F*_c_(*h*) values corresponding to all observed structure-factor amplitudes. Finally, *R*_complete_ was calculated by comparing these excluded data with the observed structure-factor amplitudes. *R*_complete_ is therefore not biased by the model, just like in standard *R*_free_ calculations, yet it is a more robust measure of model bias, especially for incomplete and/or sparse data, because all reflections contribute to its value.

## 3. Results   

### 3.1. Data integration   

**Figure 3:**
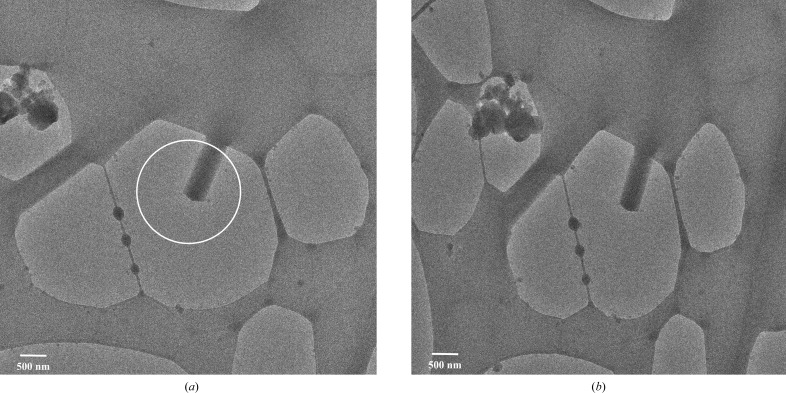
Micrographs of a single three-dimensional lysozyme crystal (200 × 500 × 1400 nm) in a thin layer of vitreous ice across a hole in the Lacey carbon EM grid at (*a*) +20° tilt angle and (*b*) +50° tilt angle. Diffraction data were acquired with a 2.0 µm diameter parallel beam in microprobe mode, indicated by a circle in (*a*). During data collection only the tip of the crystal was kept in the central beam to limit noise from the carbon support. The width of the crystal at both tilt angles was used to derive its dimensions; the length was measured from the tip of the crystal to the edge of the carbon and was the maximum size of the crystal within the central beam at any point during rotation.

Data were acquired from a single cryocooled lysozyme nanocrystal with dimensions of 200 × 500 × 1400 nm (Fig. 3 ▸). The crystal was found in a thin layer of vitreous ice over a hole in the carbon support film of the EM grid. The crystal was continuously rotated for 38.2° with an angular increment of 0.076° per frame in a 2 µm diameter beam. The central beam was positioned such that during data collection only the tip of the crystal over the hole was illuminated, thus eliminating any background noise from the amorphous carbon in the support film. In our experience, it was favourable to collect data from crystals that were still attached at one end to the carbon support. Crystal bending upon exposure to the beam was observed in cases where the crystals were suspended in vitreous ice but not attached to the carbon, probably owing to charging effects. The total dose received by the crystal did not exceed ∼4.4 e^−^ Å^−2^. Data from the single crystal were integrated to 2.1 Å resolution (Table 1 ▸, Fig. 2 ▸). The single-crystal data had a completeness of only ∼50%, but were sufficient for full structure solution (Table 1 ▸).

**Figure 4:**
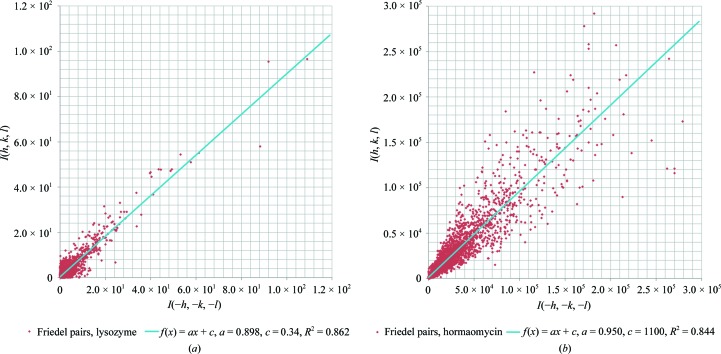
Differences in intensities of Friedel pairs after scaling plotted for (*a*) a single lysozyme crystal used for structure solution with *R*_Friedel_ = 0.329 and (*b*) X-­ray data for hormaomycin, a macrocyclic depsipeptide in space group *P*1 with *R*_Friedel_ = 0.151 (Gruene *et al.*, 2014 ▸).

To investigate the inter-crystal consistency of the data with those of other nanocrystals, we collected additional diffraction data (Table 1 ▸). After merging with diffraction data from six other nanocrystals (Supplementary Table S1) that diffracted to 2.5–3.0 Å resolution rather than 2.1 Å, the overall completeness increased to ∼60% (Supplementary Fig. S2, Supplementary Table S4). The limiting factors were radiation damage and the preferred orientation of the crystals, combined with the limited rotation range of the goniometer holding the EM grid. At higher angles, the distance that the electrons have to travel through the surrounding amorphous ice and the protein crystal can become too large for accurate data acquisition. These limitations are inherent to current implementations of electron diffraction: others have collected to up to ∼44° (Nannenga, Shi, Leslie *et al.*, 2014 ▸), ∼61° (Nannenga, Shi, Hattne *et al.*, 2014 ▸) and ∼40° (Yonekura *et al.*, 2015 ▸). These data were collected on crystals that were significantly larger than our nanocrystals, and in case of Yonekura and coworkers needed merging from 58 and 99 crystals (Table 2 ▸). Further, we compared the differences in the measured intensities of Friedel pairs after scaling but before merging of the single-crystal data set (Fig. 4 ▸). The variation in Friedel pair intensities for the single-crystal data is low, even when compared with X-ray data from a small macrocyclic depsipeptide crystal that could be solved by direct methods.

### 3.2. Structure determination   

**Figure 5:**
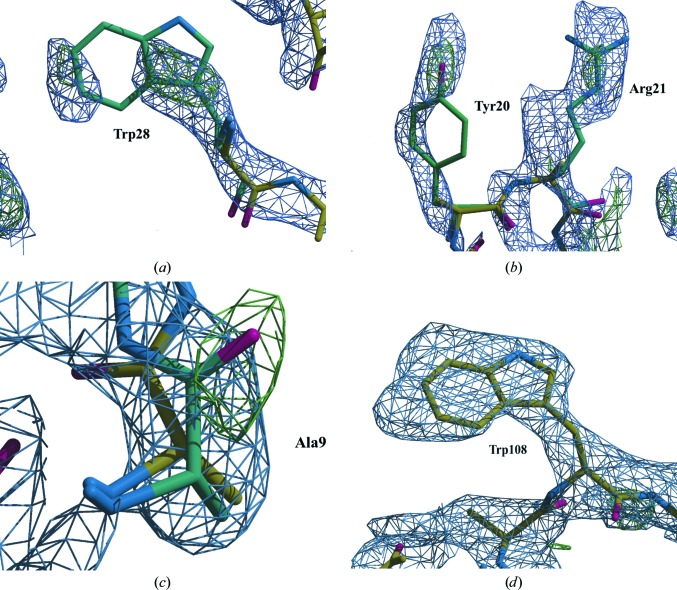
Automated model building using the single-crystal data. After molecular replacement with the polyalanine monomer (yellow C atoms), the difference map shows the position of bulky side-chain residues such as (*a*) Trp28 as placed during autobuilding by *Buccaneer* (turquoise C atoms) and (*b*) Tyr20 and Arg21. The map is stretched, which is typical for incomplete data; as always with poor map quality, careful interpretation of the region is required. The map improves after side-chain reconstruction with *Buccaneer* and refinement with *REFMAC*5. (*c*) The refined density suggests that Ala9 (yellow C atoms) is a *cis*-peptide; it is confirmed by the X-ray structure of the same polymorph (turquoise C atoms; PDB entry 4r0f) that the peptide is *cis*. Refinement using standard protocols can further improve the map and shows continuous density (*d*) for a Trp108 side-chain residue in chain *A* of the single-crystal model. All density is shown at a standard contour level of 1.2σ.

Molecular replacement with a monomeric polyalanine lysozyme model derived from a different, tetragonal space group successfully located a single monomer in the asymmetric unit. It also then placed the second monomer. A *Z*-score of 22.5 is sufficiently high above the threshold of 8.0, indicating a successful structure solution (McCoy *et al.*, 2007 ▸). Automated model building with *Buccaneer*/*REFMAC*5 (Cowtan, 2006 ▸; Murshudov *et al.*, 2011 ▸) was used to reconstruct the side chains (Figs. 5 ▸ *a* and 5 ▸ *b*). The densities that are shown were not refined. Hence, they look poor. However, they show that the molecular replacement was successful, as they demonstrate that the phases from a polyalanine MR solution allow the placement of side-chain density for atoms that were not included in the MR model. Subsequent refinement using only the observed reflections improved the quality of the map; for example, the refined density suggests that residue Ala9 is a *cis*-peptide, which differs from the tetragonal MR model (Fig. 5 ▸ *c*). However, the 1.9 Å resolution X-ray structure of the same orthorhombic polymorph confirms that the peptide is *cis* in this crystal form. This strongly validates the quality of our structure solution. Density that was refined according to standard, default protocols shows continuous, high-resolution density (Fig. 5 ▸ *d*).

At 2.1 Å resolution, and in particular with incomplete data, maps are prone to model bias. To estimate how much information our data contain, we calculated r.m.s.d. values between an X-ray model of orthorhombic lysozyme in the same space group with a similar unit cell (PDB entry 4r0f; Sharma *et al.*, 2016 ▸) and (i) our refined model with autobuilt side chains (r.m.s.d. = 0.7 Å) and (ii) our model with side-chain rotamers that are statistically preferred in proteins (r.m.s.d. = 1.1 Å) (Supplementary Table S5, Supplementary Fig. S5). This indicates that the placement of side-chain residues is based on real information contained in the single-crystal data. These results demonstrate the validity of the diffraction data, despite the relatively poor merging and model statistics compared with complete X-ray data (Table 1 ▸).

**Figure 6:**
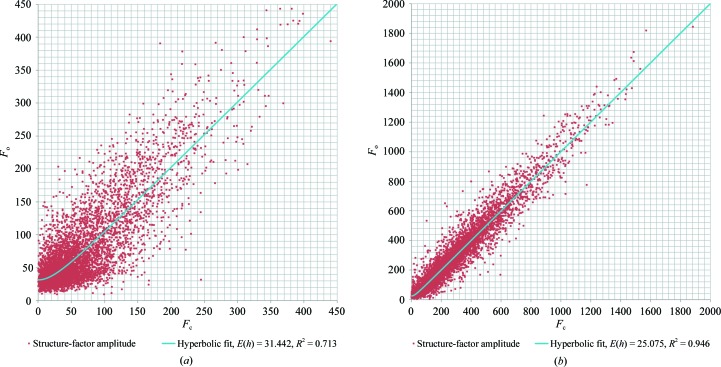
*F*_o_ *versus* *F*_c_ graphs for (*a*) electron diffraction of a single lysozyme nanocrystal and (*b*) an X-ray data set for cubic (bovine) insulin at 1.6 Å resolution. The data were least-squares fitted with a hyperbolic function described by 〈|*F*_o_|〉 = [|*F*_c_|^2^ + 〈|*E*(*h*)|〉^2^]^1/2^. *F*_o_ *versus* *F*_c_ graphs for only the low-resolution part of the single-crystal data and for the merged crystal data are shown in Supplementary Fig. S6.

To assess the influence of dynamical scattering on our electron diffraction data, we plotted *F*_o_ against the refined *F*_c_ (Fig. 6 ▸ *a*). In the absence of dynamical scattering, a linear correlation between the measured and calculated structure-factor amplitudes is expected (Fig. 6 ▸ *b*). However, in our diffraction data the correlation between *F*_o_ and *F*_c_ is no longer linear for the lower intensity structure factors. Using least squares, we fitted a hyperbolic curve to the diffraction data describing the nonlinear flattening for the lower intensity part of the *F*_o_ *versus* *F*_c_ graph. We further fitted a hyperbolic curve to the merged diffraction data, showing similar fitting parameters as found for the single-crystal data set (Supplementary Fig. S6).

## 4. Discussion   

Here, we show the structure determination from electron diffraction data of a single continuously rotated cryopreserved three-dimensional protein nanocrystal with a diffracted volume at least an order of magnitude smaller than was previously possible. For all steps of the structure elucidation, we used standard procedures and software that were originally developed for X-ray protein crystallography. The completeness of the data is low, but because there are two molecules in the asymmetric unit we could apply noncrystallographic symmetry restraints. This NCS was exploited during refinement, and the deleterious effects of data incompleteness could be mitigated. Completeness is also determined by crystallo­graphic symmetry. For instance, if the lysozyme nanocrystal had had tetragonal symmetry, instead of orthorhombic symmetry, the completeness with the same rotation range would have been 84% or greater.

Dynamical scattering has been a longstanding argument against electron crystallography of three-dimensional protein crystals. It causes the intensity of each Bragg peak to be affected by the structure factors of the other Bragg peaks that are recorded in the same exposure. When recorded in a different crystal orientation, its measured intensity will therefore be different even after scaling and Lorentz corrections. This effect also causes differences between the measured intensities of symmetry-equivalent reflections (Glaeser & Downing, 1993 ▸). Dynamical scattering can compromise structure solution of crystals of macromolecules, since current phasing methods and refinement procedures do not account for its effects. Thin crystals minimize the effects of dynamic scattering, and on the basis of multi-slice simulations it has been suggested that the maximal thickness of a protein crystal that still allows structure solution is about 100 nm for 200 keV electrons (Subramanian *et al.*, 2015 ▸), but these calculations ignore inelastic scattering, which is three times more prevalent than elastic diffraction for organic samples.

X-ray data in which the intensities of Friedel pairs correlated as poorly as in our electron diffraction data have been solved and refined using standard procedures (Fig. 4 ▸), indicating that the noise that our data suffered owing to dynamical scattering was tolerable. Furthermore, we show a *F*_o_ *versus* *F*_c_ graph of our electron diffraction data after model refinement (Fig. 6 ▸). It shows a linear correlation for the higher intensity, but at lower intensity the value of *F*_o_ is overestimated. On average, dynamical diffraction is anticipated to affect weaker reflections more than strong reflections. Thus, on average, weak spots close to intense spots will become more intense, whereas intense spots close to weak spots will hardly be affected (Weirich *et al.*, 2000 ▸). Assuming an expected complex-valued error *E*(*h*) that is uncorrelated to *F*(*h*), we can infer a hyperbolic relationship between the expected value of 〈|*F*_o_|〉 and |*F*_c_|,
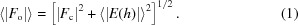



Our data indeed show such a relationship (Fig. 6 ▸). Merging reduces the random errors of the data, and should also reduce some of the dynamical effect, provided the merged crystals have different orientations. However, the fitting parameters from the *F*_o_ *versus* *F*_c_ graph for the merged data are similar compared with the single-crystal data (Supplementary Fig. S6). The expected error increases at lower resolution (Supplementary Fig. S6), indicating an increased dynamic effect within this resolution range. These observations suggest that weak spots become relatively more affected by other sources of noise with increasing resolution. Nevertheless, although the data were very weak and were compromised by dynamical scattering, they were of sufficient quality for a realistic molecular-replacement solution.

Radiation damage and the small volume of the crystal presented here severely limit the SNR and make data acquisition more challenging. We could improve the SNR substantially with a more accurate and sensitive detector. Previously, we measured three-dimensional nanocrystals similar to the polymorph presented here using CCD detectors and image plates (Georgieva *et al.*, 2007 ▸, 2011 ▸). For protein crystals that had a similar diffracting volume to that reported here, we could never measure more than a few diffraction patterns of high-resolution data with a CCD detector or image plate before radiation damage became too severe. A quantitative comparison between image plates and a Medipix hybrid pixel detector indicated a substantial improvement to be offered by the latter (Georgieva *et al.*, 2011 ▸; Nederlof *et al.*, 2013 ▸). Hybrid pixel detectors such as Medipix, Timepix and EIGER (Llopart *et al.*, 2002 ▸, 2007 ▸; Johnson *et al.*, 2012 ▸) are well suited for measuring high-energy electrons (McMullan *et al.*, 2007 ▸), and can overcome difficulties in detecting weak peaks, for example for CCD and CMOS detectors (Hattne *et al.*, 2016 ▸; Rodriguez & Gonen, 2016 ▸).

An inherent drawback of the detector design is the loss of information in the gaps between the individual tiles (each tile being a 512 × 512 quad Timepix). Because Timepix quads are connected by wire bonds to their readout electronics, these gaps are unavoidable. Without the gaps the data would have been more accurate, but not much more complete, as the geometry of the experiment allowed the data for the Friedel equivalents of most of the missing reflections to be collected (Figs. 1 ▸ and 2 ▸). The deleterious effect of the gap on data completeness can be further mitigated by aligning the rotation axis with the large gap. This would mainly lead to the loss of reflections with Lorentz factors that are so high that they would be discarded by the data-processing software anyway (Fig. 2 ▸).

The total illuminated volume of the single nanocrystal that we used for the data acquisition described here was only ∼0.14 µm^3^ (Fig. 3 ▸). The data provided sufficient information for structure solution, model building and refinement (Table 1 ▸, Fig. 5 ▸). The total diffracting volume of the crystal is no more than 6 × 10^5^ unit cells (Table 2 ▸). A comparison with structures of macromolecules previously solved by electron diffraction recorded on CCD and CMOS detectors show that these used significantly larger crystals (Nannenga, Shi, Leslie *et al.*, 2014 ▸; Nannenga, Shi, Hattne *et al.*, 2014 ▸; Yonekura *et al.*, 2015 ▸; Hattne *et al.*, 2015 ▸). Since the quality of the diffraction data from protein crystals is determined in the limiting case by the crystallinity of the sample, these data need to be interpreted with great care and should only be used to infer trends. To correct for differences in unit-cell volumes, we determined the number of unit cells used for structure solution. Resolution and crystal symmetry will also affect the amount of unique data within a data set. After correcting for these effects, the hybrid pixel detector allowed structure solution using at the very least an order of magnitude less unique diffracted intensity than obtained previously with other detectors (Table 2 ▸).

Additional hardware modifications may further benefit electron diffraction studies of macromolecular compounds, for example a reliable and well integrated goniometer tilt (Yonekura *et al.*, 2015 ▸) and using an in-column energy filter (Yonekura *et al.*, 2015 ▸). The data presented here show that with a highly sensitive and accurate hybrid pixel detector, nanometre-sized crystals of macromolecules are now also possible targets for three-dimensional protein electron crystallography, which has the advantage of reducing the effects of dynamical diffraction. It is possible that data from micrometre-sized crystals may also be measured more accurately, although it needs to be investigated further whether data accuracy is limited by detector sensitivity or the amount of dynamical diffraction for such crystals. The introduction of hybrid pixel detectors has had a major positive impact on protein X-ray crystallography owing to their high speed, increased sensitivity and high dynamic range (Broennimann *et al.*, 2006 ▸). Based on the results that we present here, we suggest that specialized hybrid pixel detectors may have a similar impact on electron diffraction studies of protein crystals.

## 5. Related literature   

The following references are cited in the Supporting Information for this article: Diederichs & Karplus (1997 ▸), van Genderen (2015 ▸), Kleywegt (1996 ▸) and Visser *et al.* (2011 ▸).

## Supplementary Material

PDB reference: lysozyme, 5o4w

PDB reference: 5o4x

Supplementary Tables and Figures.. DOI: 10.1107/S2059798317010348/tz5091sup1.pdf

## Figures and Tables

**Table 1:** Data integration and refinement statistics Values in parentheses are for the highest resolution shell; the data were truncated at *I*/σ(*I*) > 1.0 (Diederichs & Karplus, 2013 ▸).

	Single crystal (PDB entry 5o4w)	Merged data (PDB entry 5o4x)†
Data integration		
Space group	*P*2_1_2_1_2	
*a*, *b*, *c* (Å)	104.56, 68.05, 32.05	
α, β, γ (°)	90.0, 90.0, 90.0	
No. of crystals	1	7
Resolution (Å)	41.46–2.11 (2.17–2.11)	57.03–2.11 (2.17–2.11)
*R*_merge_ (%)	26.3 (56.6)	39.8 (64.0)
〈*I*/σ(*I*)〉	2.6 (1.0)	2.7 (1.0)
Completeness (%)	49.5 (49.8)	61.7 (49.8)
Reflections	12601 (1462)	41191 (1462)
Unique reflections	6749 (545)	8560 (545)
Structure solution		
Translation-function *Z*-score	22.5	26.7
LLG score	395	535
Refinement		
Reflections	6717	8503
*R*1‡ (%)	33.5	26.4
*R*_complete_ ‡ (%)	35.0	27.9
〈*B*〉 (Å^2^)	24.0	27.0
R.m.s.*Z*, bonds	0.92	0.85
R.m.s.*Z*, angles	1.27	0.97
Ramachandran plot		
Favoured (%)	93.7	98.4
Allowed (%)	5.9	1.6
Outliers (%)	0.4	0.0

† Data-integration statistics for the individual crystals used for merging are shown in Supplementary Table S1; data-merging statistics are presented in Supplementary Fig. S2 and Supplementary Table S4.

‡ We present *R*1 and *R*_complete_ instead of *R*_work_ and *R*_free_. For less than 10 000 unique reflections *R*_complete_ is preferred over *R*_free_, since it is calculated from all reflections (Brünger, 1997 ▸; Luebben & Gruene, 2015 ▸). Since all structure factors are used, this in turn leads to a more robust calculation than *R*_free_. Using this validation method, the actual refinement uses all reflections; hence, *R*_work_ is equivalent to *R*1.

**Table 2:** Relative crystal volume used for structure determination in recent macromolecular electron diffraction studies

	PDB code	Detector	*d* (Å)	Space group	Unit-cell dimensions (Å)	No. of crystals	Individual crystal size (µm) and total diffracted volume†	No. of unit cells‡ (×10^6^)	Relative unique diffracted intensity§ (×10^6^)
Lysozyme	5o4w	Hybrid pixel	2.1	*P*2_1_2_1_2	105 × 68 × 32	1	0.2 × 0.5 × 1.4 (0.14 µm^3^)	0.6	1.4
Lysozyme (Nannenga, Shi, Leslie *et al.*, 2014 ▸)	3j6k	CMOS	2.5	*P*4_3_2_1_2	76 × 76 × 37	1	0.5 × 2.0 × 2.0 (2 µm^3^)	9.4	18
Catalase (Nannenga, Shi, Hattne *et al.*, 2014 ▸)	3j7b	CMOS	3.2	*P*2_1_2_1_2_1_	68 × 172 × 182	1	0.15 × 4.0 × 6.0 (3.6 µm^3^)	1.7	14
Catalase (Yonekura *et al.*, 2015 ▸)	3j7u	CCD	3.2	*P*2_1_2_1_2_1_	69 × 173 × 206	58	0.1 × 2.0 × 2.0 (23 µm^3^)	9.4	77
Ca^2+^-ATPase (Yonekura *et al.*, 2015 ▸)	3j7t	CCD	3.4	*C*2	166 × 64 × 147 (β = 98°)	99	0.1 × 2.0 × 2.0 (40 µm^3^)	25	490

† The illuminated crystal size used for data acquisition is estimated from the reported crystal dimensions and the aperture sizes used; for the structures with PDB codes 3j7u and 3j7t (Yonekura *et al.*, 2015 ▸) we assumed that the plate-like crystals had a surface area of 2 × 2 µm. The total diffracted volume (indicated by the number in parentheses) takes the number of crystals required for the three-dimensional data set into account.

‡ The required number of unit cells was calculated by dividing the total diffracted volume by the unit-cell volume.

§ We calculated the relative unique diffracted intensity by dividing the number of required unit cells (given in the previous column) by the number of asymmetric units in the unit cell and multiplying the result by the cube of the resolution of the data set.

## Footnotes

1 Just as an example: assuming a unit cell with 100 Å axes in a well ordered protein crystal, and a preferred minimum distance of five pixels between adjacent Bragg peaks, the maximum attainable resolution for a single quad is approximately 3.5 Å if the direct beam is centred on the detector. For the same crystal, tiling four quads as presented here would increase the maximum resolution at which Bragg spots can be identified to beyond 1.0 Å, again assuming a central direct beam.
