# Supplementary material for: Protein structure determination by electron diffraction using a single three-dimensional nanocrystal
Source: Acta Crystallogr D Struct Biol. 2017 Aug 15;73(Pt 9):738–48. doi: 10.1107/S2059798317010348 (PMC5586247; doi:10.1107/S2059798317010348)
Supplement: Supplementary file 1 [file d-73-00738-sup1.pdf]

STRUCTURAL
BIOLOGY

Volume 73 (2017)

Supporting information for article:

Protein structure determination by electron diffraction using a single three-dimensional nanocrystal

M. T. B. Clabbers, E. van Genderen, W. Wan, E. L. Wiegers, T. Gruene and J. P. Abrahams

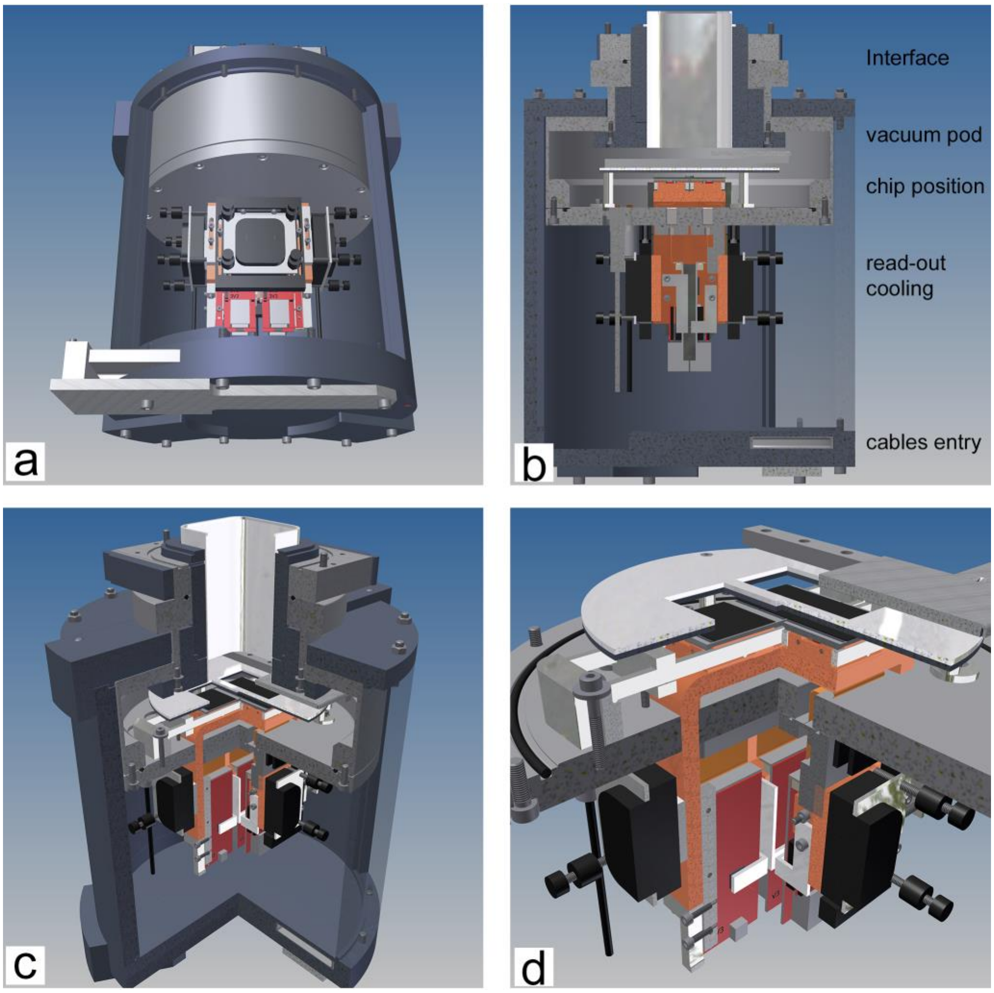

Figure S1 Design drawings of the camera showing (a) bottom to top view of the read-out and cooling of the non-vacuum part, (b) side view of a 180° cross section, (c) side view of a 90° cut-out, (d) 90° cut-out of the bottom vacuum flange showing detector chips, readout, and cooling (van Genderen, 2015). The assembly is holding four 512x512 pixel Timepix quad chips, and was developed to fit on-axis underneath a FEI Titan Krios TEM below a FEI Falcon direct electron detector. The Timepix chip assembly, read-out, and control software were provided by Amsterdam Scientific Instruments (Amsterdam, the Netherlands). The detector chips are covered by a single 300 µm thick Silicon sensor layer, allowing extraction energies of up to 200 kV on a TEM without having any significant damage to the Timepix ASICs (Faruqi & McMullan, 2011). The camera is cooled by a single water loop at four different positions from which the detector chips are being cooled (Peltier controlled) in pairs, to have a stable temperature of 2 ± 0.1 K below ambient temperature. The Relaxd read-out boards (Visser *et al.*, 2011) are directly cooled by the water loop.

Table S1 Data acquisition and integration statistics of additional crystals used for data merging

	1	2	3	4	5	6	7
**Data collection**							
Wavelength (Å)	0.02508						
Frame exposure (s)	0.5	0.5	0.2	0.3	0.3	0.3	0.3
φ_{total} (°)	38.15	42.64	20.16	20.11	20.30	20.20	20.15
Δφ (°/frame)	0.0760	0.1615	0.0344	0.0481	0.0481	0.0481	0.0481
Exposure dose (e^{-}.Å^{-2})	4.41	10.97	9.82	10.49	10.59	10.55	10.52
**Data integration**							
Space group	$P2_12_12$						
Unit cell dimensions							
a, b, c (Å)	104.56(5), 68.05(8), 32.05(3)						
α, β, γ (°)	90.0(0), 90.0(0), 90.0(0)						
Resolution (Å)[1]	41.46-2.11 (2.17-2.11)	32.05-2.50 (2.57-2.50)	41.44-3.08 (3.21-3.08)	24.41-2.54 (2.61-2.54)	27.33-2.54 (2.61-2.54)	57.04-3.06 (3.22-3.06)	52.28-3.08 (3.22-3.08)
R_{merge} (%)	26.3 (56.6)	31.7 (107.3)	19.3 (65.9)	27.5 (64.9)	25.8 (94.2)	21.1 (37.2)	24.1 (87.1)
I/σI	2.6 (1.0)	2.92 (1.10)	2.80 (1.14)	2.34 (1.09)	2.73 (1.02)	2.52 (1.25)	2.44 (1.03)
Completeness (%)	49.5 (49.8)	41.0 (40.5)	28.8 (33.1)	27.5 (28.0)	23.8 (23.5)	21.4 (15.1)	26.0 (25.0)
Reflections	12601 (1462)	9518 (817)	2040 (283)	2141 (361)	3096 (269)	1568 (150)	2092 (270)
Unique reflections	6749 (545)	3445 (236)	1326 (172)	2210 (164)	1920 (626)	1007 (104)	1199 (142)

[1] Values in parentheses correspond to the highest resolution shell, the data were truncated at I/σ > 1.0 (Diederichs & Karplus, 2013)

Table S2 Indexing with *XDS* suggests an orthorhombic lattice without prior knowledge about space group or unit cell parameters.

The quality of fit makes a large jump before the last line, correctly suggesting ‘oP’ as Bravais lattice.

**Lattice-character**	**Bravais-lattice**	**Quality of fit**	**Unit cell constants (Ångström and degrees)**					
			**a**	**b**	**c**	**α**	**β**	**γ**
31	aP	0.0	32.1	67.7	104.5	89.9	89.9	89.9
44	aP	1.9	32.1	67.7	104.5	90.2	90.1	89.9
35	mP	6.7	67.7	32.1	104.5	90.1	90.2	89.9
33	mP	14.7	32.1	67.7	104.5	90.2	90.1	89.9
34	mP	15.6	32.1	104.5	67.7	90.2	89.9	90.1
32	oP	17.5	32.1	67.7	104.5	90.2	90.1	89.9
37	mC	251.0	211.3	32.1	67.7	89.9	90.2	81.4

Table S3 *XDS* correctly suggests Laue group *mmm* from the processed data without prior knowledge about space group or unit cell parameters[1], space group number 16 autoselected by *XDS* is marked with a '*'.

Space group number		Unit cell constants (Ångström and degrees)						Unique[2]	Rmeas[3]	Compared[4]	Lattice-character	Bravais-lattice
		a	b	c	α	β	γ					
	1	32.0	67.6	104.2	89.8	89.9	89.9	876	16.3	200	31	aP
*	16	32.0	67.6	104.2	90.0	90.0	90.0	506	28.5	570	32	oP
	3	67.6	32.0	104.2	90.0	90.2	90.0	589	27.2	487	35	mP
	3	32.0	67.6	104.2	90.0	89.9	90.0	787	21.5	289	33	mP
	3	32.0	104.2	67.6	90.0	90.1	90.0	779	25.1	297	34	mP
	1	32.0	67.6	104.2	90.2	89.9	90.1	876	16.3	200	44	aP

[1] *XDS* only makes suggestions about the Laue group, not the space group.

[2] Number of unique reflections.

[3] Redundancy independent R-factor (Diederichs & Karplus, 1997).

[4] Number of reflections used for calculating Rmeas.

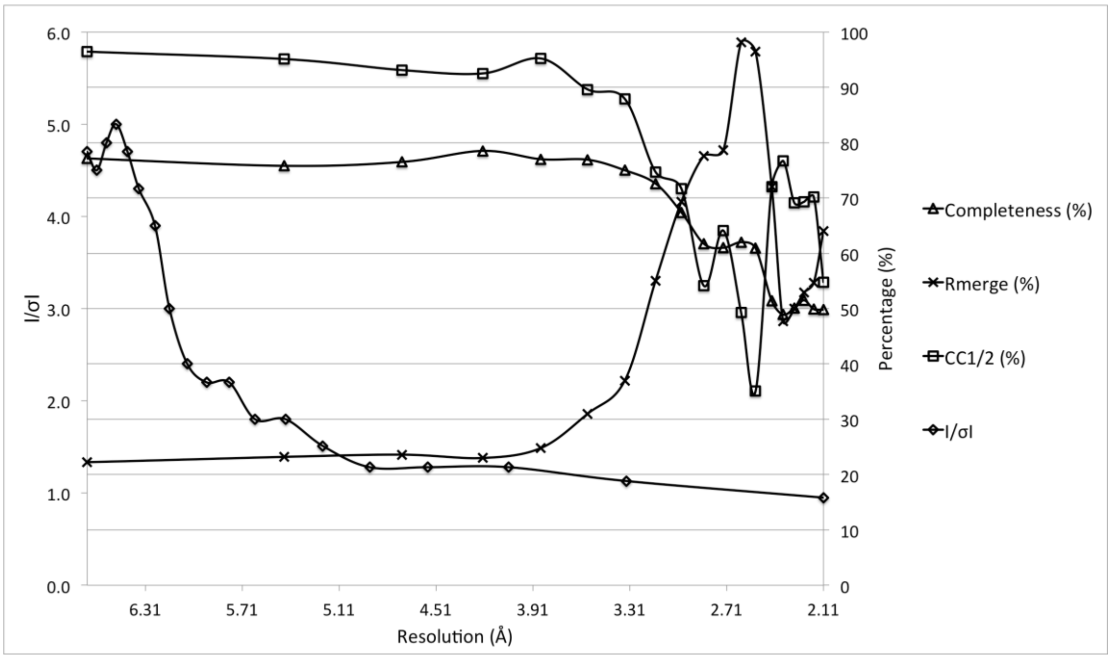

Figure S2 Data merging of seven lysozyme nanocrystals (Table 1), data completeness and data quality indicators are plotted against the resolution. Data completeness is approximately ~75% for the lower resolution bins but drops around 3.0Å and at 2.5Å since not all datasets share the same resolution cut-off (Table S1). The data were truncated at I/σI > 1.0 (Diederichs & Karplus, 2013).

Table S4 Data merging statistics from seven lysozyme nanocrystals (Table 1, Table S1) in tabular form presenting data completeness and quality indicators for each resolution bin.

The completeness over all data up to 2.1Å increases after merging from ~50% to ~60% compared to the single crystal data. The relatively low completeness can be attributed to radiation damage, preferred crystal orientation and limited goniometer rotation range.

**Resolution (Å)**	**Completeness (%)**	**Rmerge (%)**	**I/σI**	**$CC_{1/2}$ (%)**
9.43	79.4	19.8	5.3	88.6
6.67	77.1	22.2	4.7	96.4
5.45	75.8	23.2	4.5	95.1
4.72	76.5	23.6	4.8	93.1
4.22	78.5	23.0	5.0	92.5
3.86	77.0	24.8	4.7	95.2
3.57	76.9	31.0	4.3	89.6
3.34	75.0	37.0	3.9	87.9
3.15	72.6	55.0	3.0	74.7
2.99	67.4	69.3	2.4	71.7
2.85	61.7	77.6	2.2	54.1
2.73	61.0	78.6	2.2	64.1
2.62	62.0	98.1	1.8	49.3
2.53	60.9	96.4	1.8	35.1
2.43	51.4	72.0	1.5	72.0
2.36	48.9	47.7	1.3	76.7
2.29	50.1	50.0	1.3	69.1
2.23	51.5	52.9	1.3	69.3
2.17	49.9	54.7	1.1	70.2
2.11	49.8	64.0	1.0	54.8
total	61.7	39.8	2.7	91.1

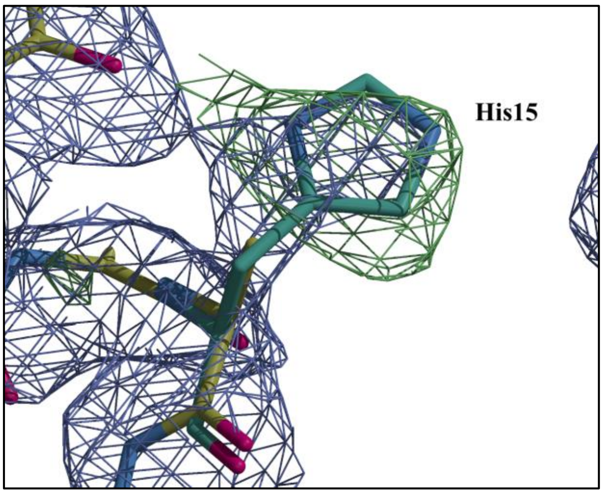

(a)

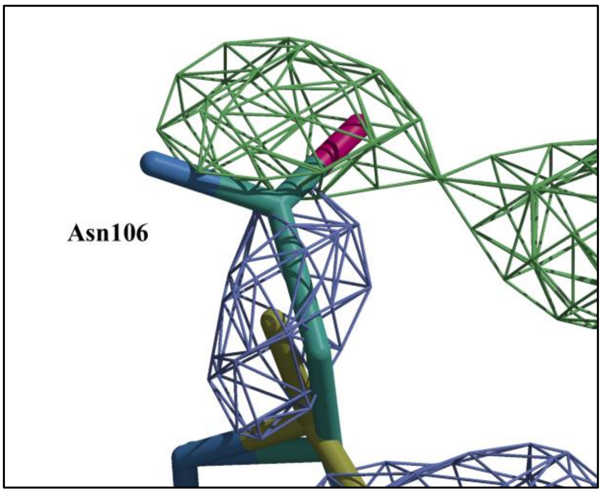

(b)

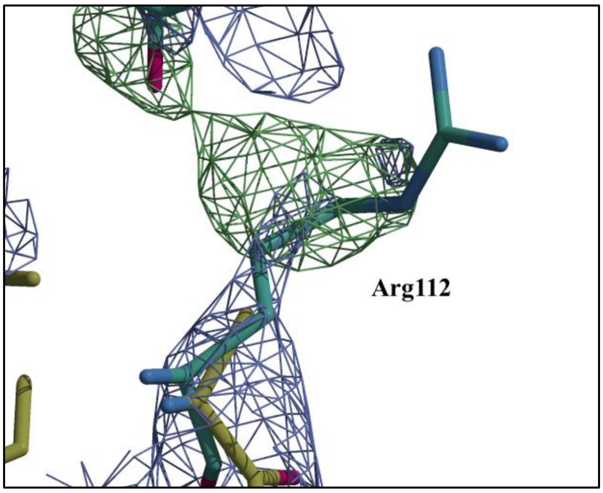

(c)

Figure S3 Electrostatic scattering potential maps of the three side chain residues that were not placed after autobuilding using the merged crystal data, albeit showing clear difference potential in favour of fitting (a) residue His15 in chain A, (b) Asn106 in chain B, and (c)

Arg112 in chain B. The yellow carbon represents the model after molecular replacement and autobuilding, the turquoise carbon model represents the fitted side chain residues. All density is shown at a standard contour level of 1.2 sigma.

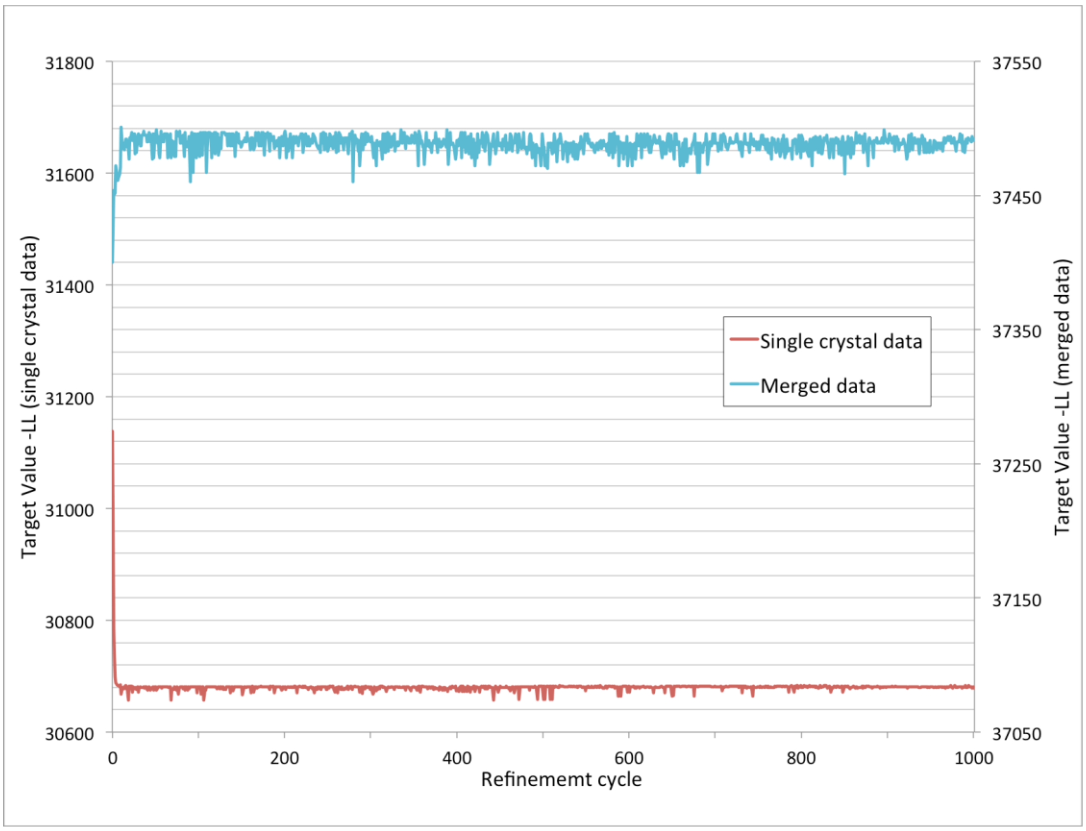

Figure S4 Model refinement with *Refmac5* for the single and merged diffraction data, indicating convergence well before the 1,000 cycles of refinement used for the final models.

Table S5 Model side chain rmsd values calculated for the single and merged lysozyme data compared to an X-ray model of orthorhombic lysozyme.

A model of the single crystal data where every side chain was replaced with the most likely rotamer shows significantly higher rmsd values indicative that during autobuilding side chains were placed based on the experimental data.

Reference data		Superimposed data		rmsd (Å)[1]
**Model**	**Chain**	**Model**	**Chain**	
2YBL	A	4R0F	A	0.479
2YBL	A	4R0F	B	0.436
Single crystal	A	4R0F	A	0.739
Single crystal	B	4R0F	B	0.829
Merged data	A	4R0F	A	0.555
Merged data	B	4R0F	B	0.597
Single crystal with most likely rotamer[2]	A	4R0F	A	1.062
Single crystal with most likely rotamer[2]	B	4R0F	B	0.982

[1] Side chain rmsd values were calculated by superimposing a X-ray model of orthorhombic lysozyme (4R0F) (Sharma *et al.*, 2016) and our model and tetragonal lysozyme (2YBL) (De La Mora *et al.*, 2011). Superposition was carried out for each residue using three main chain atoms, and rmsd values were calculated for all atoms using *LSQMAN* (Kleywegt, 1996).

[2] Here a model was created where every rotamer from our refined single crystal model was replaced by the most likely rotamer, ignoring any steric clashes.

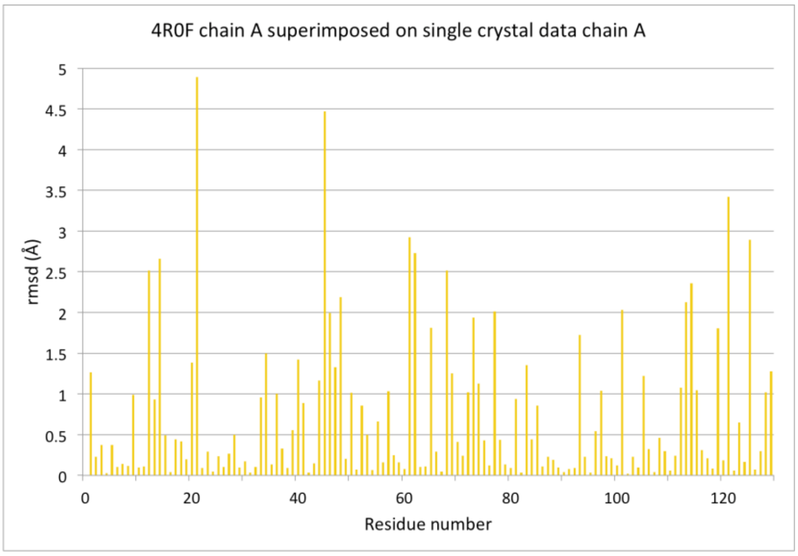

4R0F chain A superimposed on single crystal data chain A
rmsd (Å)
Residue number

(a)

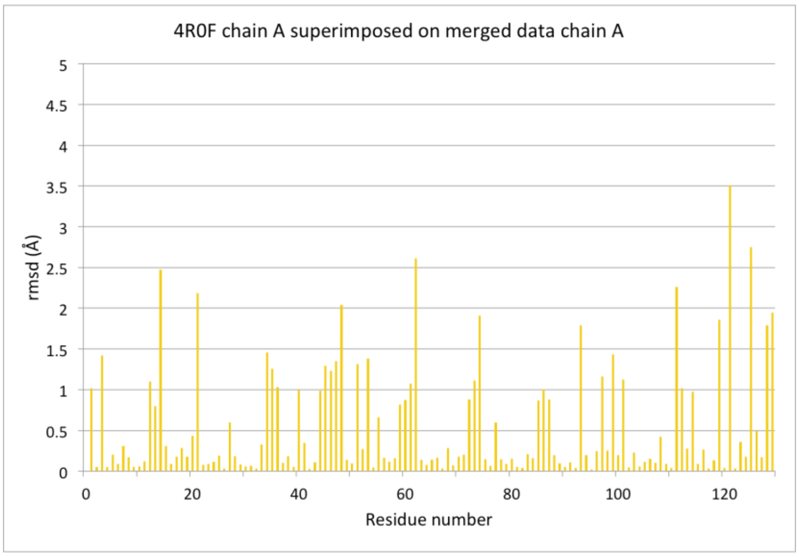

4R0F chain A superimposed on merged data chain A
rmsd (Å)
Residue number

(b)

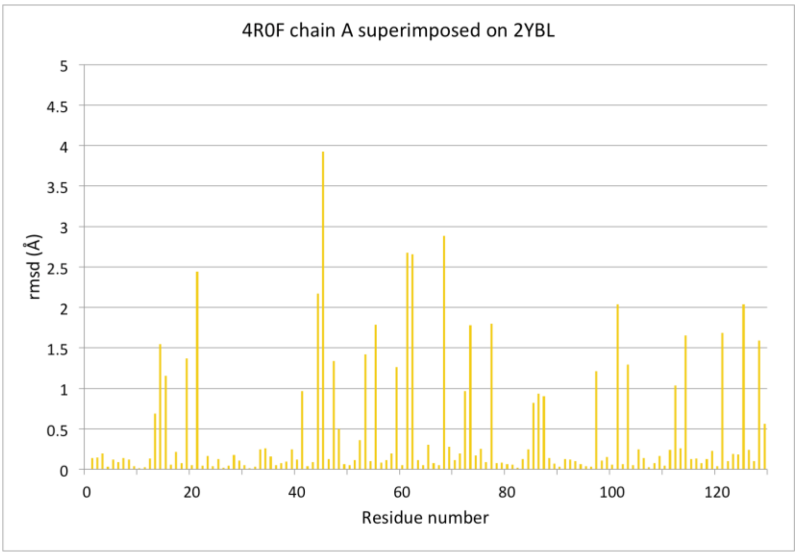

4R0F chain A superimposed on 2YBL
rmsd (Å)
Residue number

(c)

Figure S5 Side chain rmsd values plotted for each residue comparing; (a) orthorhombic lysozyme (4R0F) (Sharma *et al.*, 2016) superimposed on chain A of the single crystal data, (b) 4R0F mapped on chain A of the merged data, (c) for comparison 4R0F was superimposed on tetragonal lysozyme (2YBL) (De La Mora *et al.*, 2011), which was used for molecular replacement. Superposition and rmsd were calculated for each residue with *LSQMAN* (Kleywegt, 1996).

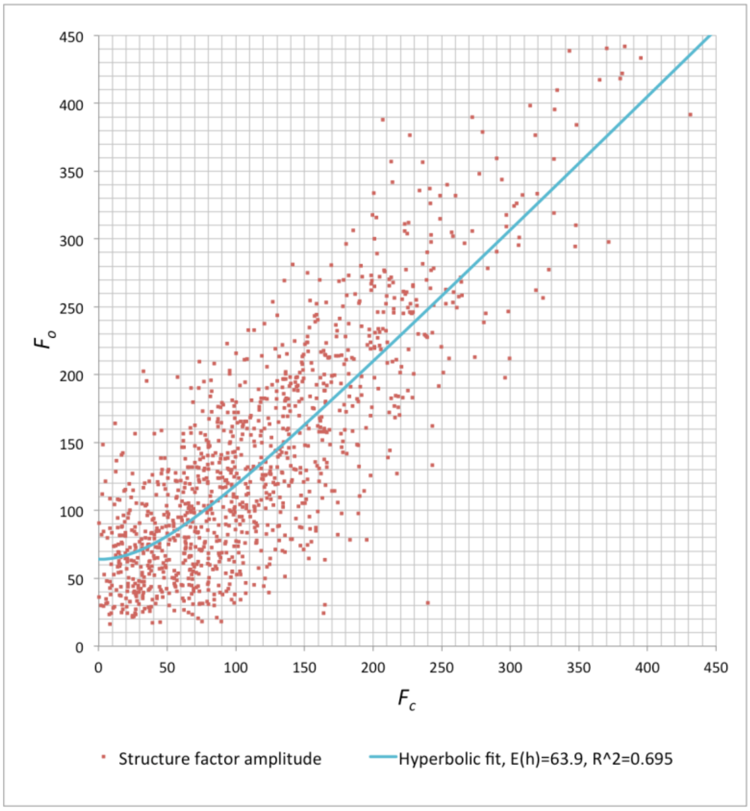

(a)

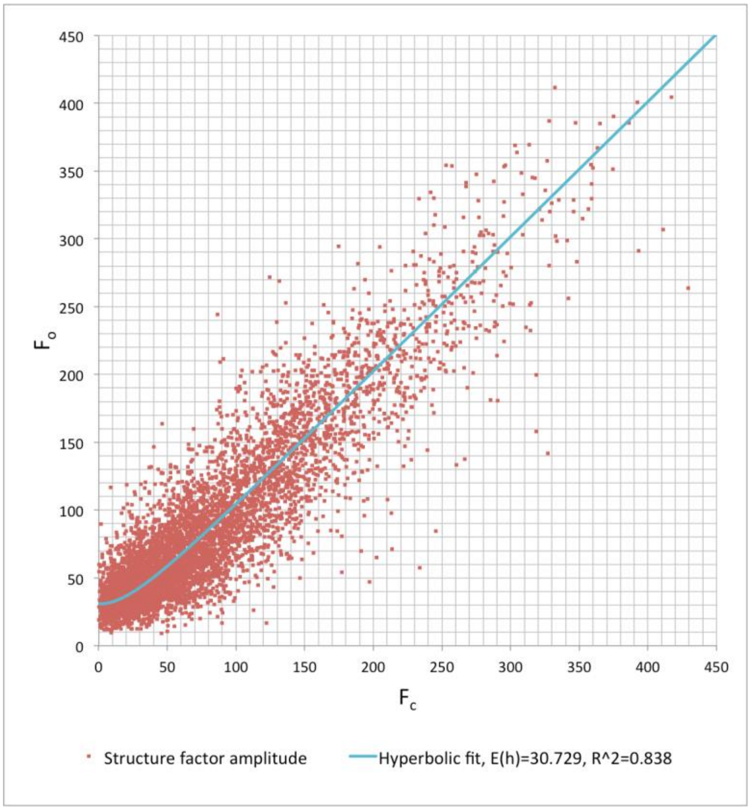

(b)

Figure S6 F_o vs. F_c graphs show the extent of dynamical scattering for (a) the low resolution single crystal data with a 4.0Å resolution cut-off, (b) the merged crystal data

obtained from merging 7 different crystals (Table S2). The data were LS fitted with a hyperbolic function described by $\langle|F_o|\rangle = \sqrt{|F_c|^2 + \langle|E(h)|\rangle^2}$.

References

Diederichs, K. & Karplus, P. A. (1997). *Nat. Struct. Biol.* **4**, 269–275.

Diederichs, K. & Karplus, P. A. (2013). *Acta Crystallogr. Sect. D Biol. Crystallogr.* **69**, 1215–1222.

Faruqi, A. R. & McMullan, G. (2011). *Q. Rev. Biophys.* **44**, 357–390.

van Genderen, E. (2015). Novel detectors and algorithms for electron nano-crystallography. Biophysical Structural Chemistry (BSC), Leiden Institute of Chemistry (LIC), Faculty of Science, Leiden University.

Kleywegt, G. J. (1996). *Acta Crystallogr. Sect. D Struct. Biol.* **52**, 842–857.

De La Mora, E., Carmichael, I. & Garman, E. F. (2011). *J. Synchrotron Radiat.* **18**, 346–357.

Sharma, P., Verma, N., Singh, P. K., Korpole, S. & Ashish (2016). *Sci. Rep.* **6**, 22475.

Visser, J., Heijden, B. Van Der, Weijers, S. J. A., Vries, R. De & Visschers, J. L. (2011). *Nucl. Inst. Methods Phys. Res. A.* **633**, S22–S25.
